# Iodine Accumulation and Biovolatilization by Filamentous Fungi and Their Effects on Extracellular Organic Acids

**DOI:** 10.3390/jof12060387

**Published:** 2026-05-28

**Authors:** Eva Duborská, Zeinab Zamani, Bence Farkas, Marek Bujdoš, Hana Vojtková

**Affiliations:** 1 Institute of Laboratory Research on Geomaterials, Faculty of Natural Sciences, Comenius University in Bratislava, Mlynská dolina, Ilkovičova 6, 84215 Bratislava, Slovakia; zamani7@uniba.sk (Z.Z.); bence.farkas@uniba.sk (B.F.); marek.bujdos@uniba.sk (M.B.); 2 Department of Environmental Engineering, Faculty of Mining and Geology, VSB—Technical University of Ostrava, 17. listopadu 2172/15, 70800 Ostrava, Czech Republic; hana.vojtkova@vsb.cz

**Keywords:** microscopic filamentous fungi, iodide, iodate, accumulation, volatilization, extracellular metabolite production

## Abstract

This study investigates the accumulation, volatilization, and metabolic effects of two iodine species (iodide and iodate) in selected filamentous fungi. Six environmentally relevant fungal strains were cultivated under controlled conditions, and iodine distribution between biomass, culture medium, and volatilized fraction was quantified using ICP-MS. Additionally, changes in extracellular metabolite production were determined by capillary isotachophoresis. The results revealed pronounced species-specific differences in iodine transformation. *Fusarium poae* exhibited the highest accumulation capacity, reaching up to 409.5 mg·kg^−1^ dry weight, and showed the most efficient overall iodine uptake from the cultivation medium. In contrast, *Alternaria tenuissima* and *Trichoderma viride* demonstrated elevated volatilization rates, particularly under iodate treatment, indicating distinct transformation pathways. Exposure to iodine species also induced significant changes in extracellular organic acids production. Increased levels of acetate, succinate, and sorbate suggest that iodine affects fungal metabolic activity, likely through stress-related shifts in central carbon metabolism and redox balance. These findings demonstrate that filamentous fungi employ diverse strategies for iodine transformation, including accumulation, volatilization, and metabolic adaptation. The results highlight the important role of fungi in regulating iodine speciation and mobility and provide new insights into their contribution to the terrestrial iodine cycle.

## 1. Introduction

Microscopic ffilamentous fungi exhibit remarkable metabolic plasticity, enabling them to transform and sequester environmental inorganic compounds through specialized metabolic pathways [1]. In the context of halogen cycling, certain species are capable of volatilizing iodine, producing compounds such as iodomethane, which represents an important pathway of iodine transformation and atmospheric emission [2,3]. Microorganisms are therefore key drivers of iodine mobility, speciation, and volatilization; however, despite their recognized importance, the underlying biochemical and molecular mechanisms remain insufficiently understood, and the available studies on microbial iodine transformation are still relatively scarce.

Fungal metabolism is highly responsive to environmental conditions and is regulated by complex networks associated with biosynthetic gene clusters (BGCs), many of which remain inactive under standard laboratory conditions [4,5,6]. Genome-based studies have revealed a vast and largely untapped biosynthetic potential in filamentous fungi, with numerous pathways activated only in response to specific environmental stimuli. Consequently, environmental factors, including trace elements and halogen availability, can act as important regulators of fungal metabolic pathways, influencing extracellular metabolite production and chemical transformation processes [7,8].

Halogen availability has been shown to significantly affect microbial metabolism, leading to the production of distinct and previously uncharacterized metabolites, while biological [3] systems are capable of transforming inorganic halides through metabolically mediated processes [8,9]. In soil environments, microbial activity further contributes to iodine mobility and speciation, thereby influencing its bioavailability and cycling. These findings suggest that iodine species may act as environmental modulators of fungal metabolism, affecting both extracellular metabolite composition and transformation pathways [10].

Fungal secondary metabolite production is tightly regulated by stress-responsive and developmental networks, enabling adaptation to changing environmental conditions and mediating interactions with other organisms, including plants [5,11]. Environmental conditions can alter the production of extracellular metabolites through regulation of biosynthetic pathways, highlighting the adaptive metabolic capacity of filamentous fungi. Variation in fungal metabolite profiles is further linked to ecological adaptation and niche differentiation, reflecting the role of secondary metabolism in environmental viability [12].

The availability of key elements, such as iron and copper, has been shown to regulate microbial secondary metabolism and activate biosynthetic gene clusters, suggesting that chemical factors can serve as signals controlling metabolic responses [13,14]. By analogy, iodine may similarly influence fungal metabolic pathways, contributing to changes in extracellular metabolite production and iodine transformation processes. The production of diverse metabolites therefore supports the ability of soil fungi to adapt to environmental conditions while influencing their surrounding chemical environment [15].

Taken together, these findings highlight the importance of environmentally regulated fungal metabolism in controlling iodine transformation, while emphasizing that many of the underlying biochemical and regulatory mechanisms remain insufficiently understood. Studies specifically addressing fungal-mediated iodine accumulation, volatilization, and associated metabolic responses are still limited. Therefore, the aim of this study was to investigate the accumulation and volatilization of iodine by selected microscopic filamentous fungi and to evaluate the associated changes in extracellular metabolite production.

## 2. Materials and Methods

### 2.1. Chemicals and Reagents

Potassium iodide (KI) and potassium iodate (KIO_3_) (p.a., Centralchem, Bratislava, Slovakia) were used to prepare iodide (I^−^) and iodate (IO_3_^−^) stock solutions. Stock solutions (500 mg·L^−1^ as iodine) were prepared in deionized water under aseptic conditions. For cultivation experiments, 1 mL of the stock solution was added to the growth medium to obtain a final iodine concentration of 10 mg·L^−1^.

### 2.2. Fungal Strains

Six distinct strains of filamentous fungi were selected based on their environmental prevalence in soils and their association with plant roots. The strains were obtained from the Culture Collection of Microorganisms (Czech Collection of Microorganisms (CCM), Department of Experimental Biology, Masaryk University, Brno, The Czech Republic) and are listed in Table 1.

### 2.3. Fungal Cultivation and Experimental Setup

Fungal colonies were cultured and maintained on Sabouraud agar slants (4% *w*/*v*; HiMedia, Mumbai, India) at 25 °C. For bioaccumulation experiments, 100 mL Erlenmeyer flasks containing 50 mL of Sabouraud dextrose broth (3% *w*/*v*; HiMedia, Mumbai, India) were used. The growth medium was sterilized by autoclaving at 120 °C for 20 min prior to inoculation. Fungal suspensions were prepared from 7-day-old cultures.

Cultivation was carried out under three experimental conditions: (i) control without iodine addition, (ii) medium supplemented with 10 mg·L^−1^ KI, and (iii) medium supplemented with 10 mg·L^−1^ KIO_3_. Each condition was prepared in triplicate. The cultures were incubated in the dark at 25 °C for 14 days in incubation chamber (Pol-Eko, Wodzisław Śląski, Poland) After incubation, the biomass was separated from the growth medium, rinsed with deionized water, dried at room temperature, and weighed. The spent growth medium was filtered through a 0.45 μm mixed cellulose ester (MCE) membrane filter (Advantec MFS, Dublin, CA, USA), and pH was measured (Hanna Instruments, Padova, Italy).

### 2.4. Iodine Determination

Iodine concentrations in the spent cultivation media were determined immediately after cultivation and filtration by inductively coupled plasma mass spectrometry (Thermo Scientific iCAP Q, Waltham, MA, USA). Prior to analysis, tetramethylammonium hydroxide (TMAH) (Alfa Aesar, Kandel, Germany) was added to achieve a final concentration of 1% (*v*/*v*) in order to stabilize iodine species.

Iodine in fungal biomass was determined after drying the samples to constant weight. Subsequently, iodine was extracted using 25% (*w*/*v*) tetramethylammonium hydroxide at 90 °C in a thermostatic bath (Biosan, Riga, Latvia). Extractions were performed in centrifuge tubes using 0.25 g of dried biomass.

The volatilization of iodine was estimated as the difference between the initial iodine concentration and the total iodine recovered in the fungal biomass and the cultivation medium after incubation.

The extracts were diluted to a final TMAH concentration of 1% (*v*/*v*) prior to analysis by ICP-MS. The operating parameters of the ICP-MS are summarized in Table 2.

### 2.5. Determination of Low-Molecular-Weight Organic Acids (Fungal Extracellular Metabolites)

Low-molecular-weight organic acids in the spent cultivation media were determined by capillary isotachophoresis (c-ITP). Anion analysis was performed using 10 mL of culture medium filtrate (0.45 μm), collected immediately after 14 days of cultivation. Measurements were carried out using a ZKI 01 isotachophoretic analyser (Villa Labeco, Spišská Nová Ves, Slovakia) operating in ITP–ITP mode. The leading electrolyte (solution A5) consisted of 10 mM HCl, 15 mM ε-aminoc aproic acid (ε-ACA), 0.1% hydroxyethyl cellulose (HEC), and 5 mmol·L^−1^ caproic acid adjusted with histidine. The terminating electrolyte (TE 5 and TE 2) containing caproic acid with histidine supplied by the manufacturer (Villa Labeco, Spišská Nová Ves, Slovakia).

For the determination of citric acid, a separate leading electrolyte (solution A2) was used, consisting of 10 mM HCl, 12 mM β-alanine (β-ALA), and 0.1% methylhydroxyethyl cellulose (MHEC), while the terminating electrolyte remained unchanged.

Quality Control Standard (QCS) QCS were prepared from acetic acid, citric acid, gluconic acid, itaconic acid, lactic acid, malic acid, oxalic acid, sorbic acid, succinic acid, and tartaric acid (Merck, Darmstadt, Germany). 100 μM stock solution of each were diluted to obtain 0.1 mM standard solutions which were analyzed prior to each Quantification of organic acids was performed using external calibration with these standard solutions. Data acquisition and evaluation of isotachophoregrams were performed using ITPpro 32 software, version 1.0.4 rev 26 provided by Villa Labeco, Spišská Nová Ves, Slovakia.

For the determination of these solutions doubled distilled water (DDW) water was used.

### 2.6. Statistical Analysis

All experiments were performed in triplicate, and results are presented as mean ± standard deviation. Quantification of organic acids was based on external calibration curves prepared from standard solutions. Calibration curves were constructed by plotting zone length against known concentrations of individual organic acids.

Statistical analysis was performed using Microsoft Excel. Differences between experimental conditions were evaluated using Student’s *t*-test, with statistical significance considered at *p* < 0.05. Although the number of biological replicates was limited (*n* = 3), the test was applied due to its relative robustness in balanced experimental designs with small sample sizes [16].

## 3. Results and Discussion

### 3.1. Toxic Effect of Iodine Species on Biomass Production and pH

The most evident fungal characteristics reflecting physiological responses to iodide and iodate during cultivation are changes in biomass production and culture medium pH. Growth parameters are commonly used for toxicity evaluation [17], whereas pH is often overlooked in studies dealing with potentially toxic elements. However, pH variations may reflect alterations in nutrient uptake, as membrane-bound ATP-driven proton pumps play a key role in maintaining the electrochemical gradient required for transport processes [18].

**Figure 1:**
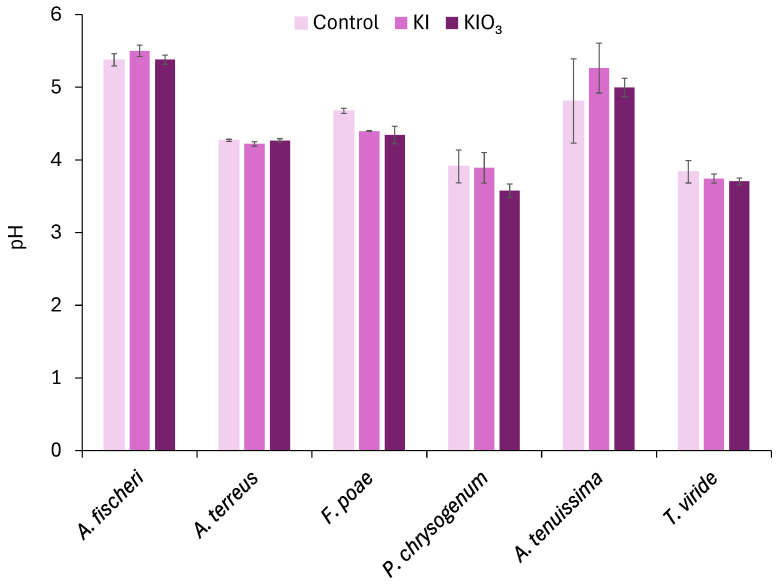
Changes in pH values after 14-day cultivation of selected fungal strains.

**Figure 2:**
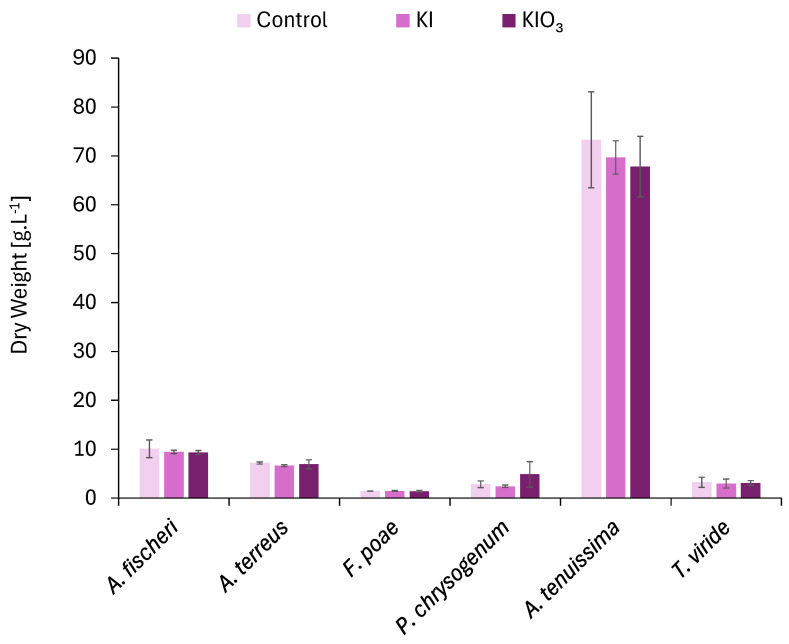
Changes in fungal biomass (dry weight) after 14 days of cultivation of selected filamentous fungal strains expressed in g·L^−1^ culture media.

In the present study, no statistically significant differences (*p* < 0.05) in final pH values (Figure 1) or dry biomass weight were observed between the control and iodine-treated cultures (iodide or iodate) across all investigated fungal strains (Figure 2). The final pH values remained comparable under all experimental conditions, indicating that neither iodine species caused significant changes in fungal metabolic activity affecting medium acidification or alkalization at the applied experimental concentrations. Similarly, biomass production was not significantly affected, suggesting that the applied iodine concentrations did not exert significant adverse effects on fungal growth under the experimental conditions used in this study.

### 3.2. Fungal Iodine Accumulation, Volatilization and Removal Efficiency

**Figure 3:**
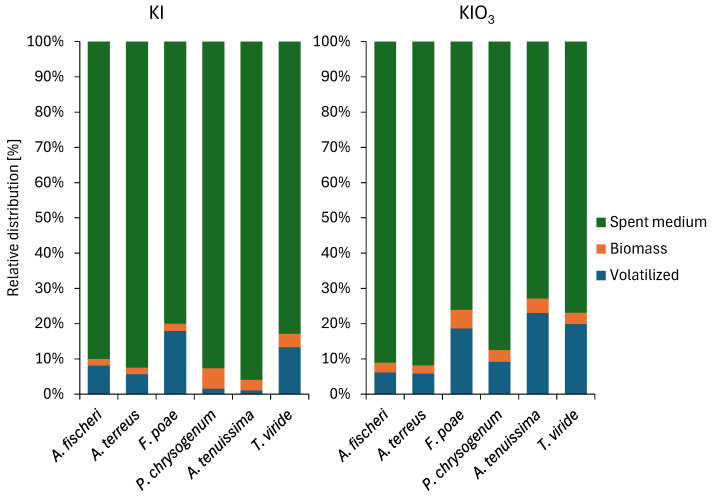
Percentual distribution of accumulated iodine, iodine remaining in the cultivation medium, and volatilized iodine.

Among the investigated fungal strains, *F. poae* exhibited the highest iodine accumulation, reaching 5.9% and 5.3% of the initial iodine from KI- and KIO_3_-treated samples, respectively (Table 3; Figure 3). This species also showed substantial iodine removal from the culture medium, with 23.2% (KI) and 23.9% (KIO_3_). When normalized to biomass, *F. poae* was the most efficient accumulator, with iodine concentrations of 409.5 mg·kg^−1^ (KI) and 378 mg·kg^−1^ (KIO_3_). The iodine content in biomass, spent cultivation medium and the amount of volatilized iodine is summarized in Table 3. Percentual distribution is illustrated in Figure 3.

The high iodine removal efficiency observed in *F. poae* and *A. tenuissima* (up to 27.2% from KIO_3_) underscores the significant role of filamentous fungi in regulating iodine fluxes in environmental systems. The accumulation capacity of *F. poae* is markedly higher than previously reported values for other environmental fungi, such as *A. alternata* [10], highlighting the species-specific nature of iodine uptake.

These results suggest that *F. poae* possesses an efficient mechanism for iodine uptake, likely driven by active transport or surface adsorption processes. The relatively high iodine content in the biomass compared to total removed iodine from the culture medium indicates that accumulation represents a dominant pathway for this species [10,19]. The strains of *A. tenuissima* and *T. viride* also demonstrated high removal efficiencies, particularly under iodate treatment, reaching 27.2% and 23.1%, respectively (Table 3). However, the proportion of iodine determined in their biomass was comparatively lower, suggesting that additional transformation pathways contribute to iodine removal.

Overall, the results demonstrate distinct, species-specific strategies of iodine transformation among filamentous fungi. While *F. poae* primarily removes iodine through accumulation, other species appear to rely on a combination of processes, indicating functional diversity in fungal responses to iodine exposure.

In this study the results highlight a strong volatilization capacity in *A. tenuissima* and *T. viride*, reaching 23.0% and 19.9% of the initial iodine, respectively, particularly under iodate treatment (Figure 3). However we did not have abiotic control in this study, the biological origin of iodine mobilization is supported by the absence of significant iodine loss in sterile, non-inoculated control media in our previous study [10]. Moreover, iodine biovolatilization appears to be strongly strain-dependent; for example, *A. alternata* exhibits high volatilization efficiency, whereas *P. citrinum* shows no detectable volatilization activity, likely relying on alternative detoxification mechanisms such as iodide efflux [10]. However, cannot be excluded, that the total iodine loss may include a minor abiotic component.

These findings are consistent with previous studies demonstrating that filamentous fungi are significant contributors to the production of volatile organic iodine compounds, especially methyl iodide (CH_3_I) [2,10,19,20].

The higher volatilization rates observed in iodate-treated samples compared to iodide treatments suggest that iodine may undergo a reductive transformation (IO_3_^−^ → I^−^) prior to methylation. Such a pathway has been proposed in microbial systems, where reduced iodine species serve as substrates for enzymatic methylation [21]. Previous studies suggest that iodate-mediated biovolatilization may proceed through an initial microbial reduction in iodate (IO_3_^−^) to iodide (I^−^), followed by enzymatic methylation of the reduced iodine species into volatile methylated compounds such as CH_3_I. Microbial iodate reduction has been described as a biologically mediated process involving enzymatic pathways and redox-active extracellular metabolites. Subsequently, iodide may serve as a substrate for methyltransferase-mediated biomethylation according to the Challenger-type mechanism or related enzymatic pathways. In addition, previous studies on filamentous fungi indicated that iodate may represent a preferable precursor for iodine biovolatilization under terrestrial conditions. Therefore, the higher biovolatilization rates observed in iodate-treated samples in the present study may be associated with microbial iodate reduction followed by subsequent methylation of the produced iodide species [19,20].

This biovolatilization process represents a key step in the global iodine cycle, facilitating the transfer of iodine from terrestrial and aquatic environments into the atmosphere [22,23]. The observed species-specific differences further indicate that volatilization is not a universal fungal trait but rather a regulated metabolic process dependent on physiological and environmental factors.

To contextualize the observed iodine transformation capacities, a comparison of accumulation and volatilization among selected fungal species from this study and previous works is presented in Table 4.

### 3.3. Alterations in Extracellular Metabolite Production

Changes in extracellular metabolite production in response to iodine species are shown in Table 5. Significant alterations were observed in the production of selected low-molecular-weight organic acids across the investigated fungal strains.

*A. fischeri* and *P. chrysogenum* produced significantly higher amounts of acetate in KI-treated samples compared to the control (*p* < 0.05), indicating a shift in central carbon metabolism. Acetate may serve both as a metabolic byproduct of stress and as a precursor for secondary metabolite synthesis, reflecting alterations in glycolytic pathwaysSimilarly, *A. terreus* exhibited a statistically significant increase in succinate and sorbate production under iodine treatment. Succinate, a key intermediate of the tricarboxylic acid (TCA) cycle, may accumulate due to metabolic redirection or bottlenecks under stress conditions. These organic acids can also function as chelating and redox-active agents, likely affecting iodine speciation, bioavailability, and toxicity in the surrounding medium [24,25].

ND has now been defined in Table 5 as “Not detected”. In the present study, compounds were considered not detected when no corresponding isotachophoretic zone was observed or when the detected zone migration time was shorter than 1 s.

The metabolic response appeared more pronounced under iodide treatment for certain species, likely due to the higher chemical reactivity of iodide compared to the more stable iodate [19]. This evidence indicates that iodine not only undergoes transformation by fungal activity but also actively modulates fungal metabolic processes. The variability in metabolite secretion profiles suggests that fungal species employ distinct metabolic strategies to cope with iodine-induced stress, reflecting changes in carbon allocation and redox balance as adaptive mechanisms for maintaining cellular homeostasis [26,27].

Extracellular metabolites may additionally influence the surrounding microenvironment by modifying pH, complexing inorganic species, and affecting iodine mobility. Increased production of low-molecular-weight organic acids may also facilitate interactions with other microorganisms and support co-metabolic processes involved in iodine transformation within the hyphosphere [28].

The production of gluconate in the extracellular environment is typically associated with glucose oxidation mediated by glucose oxidase, a pathway strongly regulated by environmental conditions, oxygen availability, and carbon source [29]. Differences in gluconate production among fungal species observed in this study, therefore, likely reflect differences in carbon metabolism and extracellular oxidative pathways. In this study, *A. terreus* produced approximately 18% less gluconate in the presence of iodide, suggesting a redistribution of carbon flux toward intracellular metabolic pathways involved in stress response and cellular maintenance. Similar metabolic reprogramming under stress conditions has been observed in fungal interaction and environmental stress studies [24,30]. In contrast, *P. chrysogenum* produced higher amounts of gluconate in the presence of both iodine species, which may represent an adaptive mechanism for environmental modification, detoxification, or pH regulation. *A. tenuissima* showed increased gluconate production in iodide treatments but decreased production in iodate treatments, indicating that iodine speciation influences metabolic adjustments differently across species. Such links between carbon metabolism and iodine transformation processes have been observed in microbial systems where iodine speciation is strongly influenced by microbial metabolic activity and carbon substrate utilization [31].

The increased production of acetate and lactate observed in some fungal strains suggests a shift in central carbon metabolism toward fermentative or overflow metabolic pathways under iodine stress. Such metabolic adjustments are commonly associated with stress conditions affecting mitochondrial function, redox balance, or energy production, forcing cells to rely more heavily on substrate-level phosphorylation and alternative metabolic pathways for ATP generation [32,33,34]. Conversely, decreased acetate and lactate production in some species may indicate a redirection of carbon flux toward alternative metabolic pathways or more efficient aerobic metabolism. These species-specific metabolic responses highlight the diversity of fungal stress adaptation strategies [35].

Changes in intermediates of the tricarboxylic acid cycle, such as malate, succinate, citrate, and oxalate, indicate broader metabolic reprogramming under iodine stress. The extracellular accumulation of these organic acids suggests overflow metabolism or metabolic rebalancing, allowing fungi to regulate intracellular metabolite concentrations, maintain redox balance, detoxify the cell, and modify the surrounding chemical environment [36,37]. Citrate production decreased in *P. chrysogenum* and *T. viride* under iodine treatment, suggesting redirection of carbon flux toward alternative metabolic pathways such as the glyoxylate cycle or other stress-response pathways [38,39]. In contrast, *F. poae* and *A.s terreus* showed changes in malate, oxalate, and succinate production, indicating species-specific adaptations in central carbon metabolism and possible involvement of the glyoxylate cycle and associated metabolic pathways [40,41,42].

Oxalate production is a well-established fungal strategy for metal detoxification and nutrient acquisition, as oxalate can chelate metal ions and form insoluble metal–oxalate complexes, consequently reducing metal toxicity and influencing metal mobility in the environment [43,44]. Organic acids such as oxalate, citrate, malate, and gluconate, therefore, not only reflect metabolic adaptation to iodine-induced stress but may also influence iodine speciation, mobility, and bioavailability in the surrounding environment [43].

Filamentous fungi commonly secrete organic acids under conditions of nutrient limitation or excess carbon availability, a phenomenon recognized as overflow metabolism, in which carbon uptake exceeds the capacity for biomass formation and respiratory metabolism, resulting in extracellular accumulation of organic acids [45]. The strong secretory capacity of filamentous fungi and their ability to release extracellular metabolites play an important role in environmental interactions, nutrient acquisition, detoxification, and stress adaptation [46]. The increased production of organic acids observed in this study, therefore, likely indicates both overflow metabolism and stress-induced metabolic reprogramming triggered by iodine exposure.

Furthermore, iodate may potentially interfere with phosphorus uptake due to structural similarities between iodate and other oxyanions transported via anion transport systems. Such interactions could impair phosphorus assimilation, which is essential for ATP synthesis and central metabolic functions [47]. Under nutrient limitation or metabolic stress, fungi often activate alternative metabolic pathways such as the glyoxylate cycle to conserve carbon and preserve energy balance [48,49].

To summarize, the observed changes in extracellular metabolite production indicate that exposure to iodine species induces broad metabolic reprogramming in filamentous fungi, affecting central carbon metabolism, organic acid secretion, fermentation pathways, and potentially nutrient uptake processes. The observed effects are likely indirect and associated with iodine transformation processes within fungal cells, where iodide and its conversion into more redox-active iodine species may induce oxidative stress and disturb intracellular redox homeostasis. The differences observed in the composition and concentrations of extracellular low-molecular-weight organic acids between iodine-treated samples and controls further suggest alterations in central carbon metabolism associated with fungal stress adaptation. These metabolic changes represent stress-adaptation mechanisms and may also significantly influence iodine speciation, mobility, and volatilization in the environment.

The combined results demonstrate that filamentous fungi exhibit distinct, species-specific strategies for coping with iodine exposure. While *F. poae* showed the highest accumulation capacity, *A. tenuissima* and *T. viride* were characterized by enhanced volatilization, indicating different dominant transformation pathways. A limitation of the present study is that the experiments were performed under relatively short-term static cultivation conditions, which may not fully reflect long-term fungal adaptation to iodine exposure or the influence of dynamic environmental factors. Prolonged cultivation under static conditions is constrained by nutrient depletion and accumulation of metabolic products, potentially affecting fungal viability and metabolic activity. In addition, substantial differences in biomass production were observed among the investigated fungal strains, suggesting species-specific nutritional requirements and physiological responses to the selected cultivation conditions. Interestingly, the highest iodine accumulation efficiency was observed in *F. poae* despite its relatively low biomass production, indicating that iodine accumulation capacity may not be directly associated with fungal growth intensity and may instead reflect stress-related adaptive mechanisms. Future studies should therefore employ continuous-flow or bioreactor-based systems enabling controlled nutrient supply, oxygen availability, pH regulation, and long-term monitoring of iodine transformation processes under environmentally relevant conditions. Such approaches may help clarify the relationship between fungal growth conditions, biomass production, and iodine accumulation efficiency.

## 4. Conclusions

This study demonstrates that filamentous fungi exhibit distinct, species-specific strategies for iodine transformation, including accumulation, volatilization, and metabolic adaptation. *F. poae* was identified as the most efficient accumulator of iodine, while *A. tenuissima* and *T. viride* showed higher volatilization capacities, indicating different dominant transformation pathways among the investigated species.

The observed changes in extracellular metabolite production indicate that iodine exposure induces measurable metabolic responses and imposes a metabolic cost, leading to the reorganization of central metabolic pathways. These adjustments likely represent integrated stress-response mechanisms that balance detoxification, energy metabolism, and environmental interaction. Iodine transformation processes may indirectly affect fungal metabolic pathways through disturbances in intracellular redox homeostasis and oxidative stress induced by redox-active iodine species. Such stress-related effects may subsequently influence central carbon metabolism and extracellular organic acid production. The metabolic shifts observed in organic acid production may therefore influence iodine speciation, mobility, and bioavailability in the surrounding environment.

The coupling of iodine transformation processes with changes in extracellular metabolite profiles suggests that fungi actively participate in regulating iodine speciation and mobility in environmental systems. Through combined processes of accumulation, biovolatilization, and metabolic exudation, filamentous fungi contribute to the redistribution of iodine between solid, dissolved, and gaseous phases, highlighting their important role in the biogeochemical cycling of iodine. The observed species-specific differences in metabolite profiles and iodine transformation efficiency may reflect variability in iodine uptake, iodate reduction, methylation capacity, extracellular metabolite secretion, and tolerance mechanisms among the investigated fungal strains.

From an ecological perspective, these processes may influence nutrient cycling, microbial community interactions, and the overall functioning of soil systems. The ability of fungi to modulate both iodine chemistry and the surrounding metabolome highlights their role as key drivers of biogeochemical transformations in iodine-affected environments. These findings improve our understanding of fungal-mediated iodine dynamics and suggest potential applications in bioremediation and biofortification strategies.

The complexity of these interactions indicates that future research should focus on linking metabolic responses with underlying genetic and regulatory mechanisms. Advanced approaches such as transcriptomics, metabolomics, proteomics, and other multi-omics techniques will be essential for elucidating the pathways governing iodine transformation, extracellular metabolite production, and the role of fungal metabolic plasticity in iodine cycling and broader soil biogeochemical processes. However, since molecular-level pathways were not directly investigated in the present study, the proposed mechanisms should be considered as plausible interpretations requiring further experimental verification.

## Figures and Tables

**Table 1:** Microscopic filamentous fungal strains used in this study.

Name	Designation of Strain
*Aspergillus fischeri*	CCM 8232
*Aspergillus terreus*	CCM F-413
*Fusarium poae*	CCM 8313
*Penicillium chrysogenum*	CCM F-362
*Alternaria tenuissima*	CCM 8212
*Trichoderma viride*	CCM F-486

**Table 2:** ICP-MS operating conditions for ^127^I determination (standard mode).

**Sample Introduction**	
Sample uptake	0.40 mL·min^−1^
Nebulizer type	Concentric PFA µFlow
Spray chamber	Cyclonic Quartz
Internal standard	^126^Te 30 µg·L^−1^
Sample background	0.5% TMAH
**ICP**	
RF power	1550 W
Plasma gas	Argon, 14 L·min^−1^
Auxiliary gas	Argon, 0.8 L·min^−1^
Cones	Nickel
**Data Acquisition**	
Acquisition mode	Peak hopping
Dwell time	50 ms
Integration time	1000 ms
Limit of detection	0.01 µg·L^−1^
Measurement uncertainty	±1.5%

**Table 3:** Iodine accumulation in fungal biomass, volatilization, and removal efficiency from the cultivation medium from initial 10 mg·L^−1^.

Fungal Species	Iodine Species	Accumulation in Biomass [mg·kg^−1^]	Biovolatilization [mg]	Remaining Iodine in Spent Culture Media [mg·L^−1^]
*A. fischeri*	KI	20.1 ± 4.1	0.041 ± 0.018	9.07 ± 0.36
	KIO_3_	29.8 ± 6.2	0.031 ± 0.028	9.05 ± 0.50
*A. terreus*	KI	30.3 ± 5.3	0.028 ± 0.004	9.30 ± 0.05
	KIO_3_	32.9 ± 5.1	0.029 ± 0.005	9.13 ± 0.11
*F. poae*	KI	409.5 ± 40.3	0.086 ± 0.005	7.74 ± 0.12
	KIO_3_	379.7 ± 61.9	0.093 ± 0.013	7.56 ± 0.24
*P. chrysogenum*	KI	117.7 ± 21.9	0.008 ± 0.006	8.92 ± 0.12
	KIO_3_	80.9 ± 33.4	0.046 ± 0.025	8.66 ± 0.50
*A. tenuissima*	KI	5.2 ± 1.3	0.005 ± 0.003	8.89 ± 0.12
	KIO_3_	6.1 ± 1.4	0.114 ± 0.083	7.21 ± 1.70
*T. viride*	KI	97.9 ± 20.2	0.063 ± 0.023	7.81 ± 0.46
	KIO_3_	105.2 ± 34.4	0.099 ± 0.055	7.62 ± 1.05

**Table 4:** Comparison of iodine accumulation and volatilization among selected filamentous fungi from this study and previous studies.

Species	Iodine Species	In Solution [%]	Biomass [%]	Volatilized [%]	Reference
*A. alternata*		39.0	49.0	0.07	[2]
*A. alternata*	KI	51.8	37.0	11.2	[10]
	KIO_3_	48.6	32.7	18.7	
*A. tenuissima*	KI	95.1	3.8	1.1	This study
	KIO_3_	72.8	4.2	23.0	
*A. clavatus*	KI	90.0	8.6	1.4	[10]
	KIO_3_	86.2	7.9	6.1	
*A. fischeri*	KI	90.0	1.9	8.1	This study
	KIO_3_	91.0	2.8	6.2	
*A. niger*		86.0	0.92	0.06	[2]
*A. oryzae*		88.0	1.0	0.07	[2]
*A. terreus*	KI	92.4	2.0	5.6	This study
	KIO_3_	91.8	2.3	5.9	
*C. cladosporioides*	KI	89.0	3.9	7.1	[10]
	KIO_3_	81.6	2.9	11.5	
*C. cladosporioides*		53.0	37.0	0.12	[2]
*F. oxysporum*	KI	84.4	9.0	6.6	[10]
	KIO_3_	80.9	8.9	10.2	
*F. poae*	KI	76.8	5.9	17.2	This study
	KIO_3_	76.1	5.3	18.7	
*P. chrysogenum*		90.0	0.34	0.23	[2]
*P. chrysogenum*	KI	95.4	3.0	1.6	This study
	KIO_3_	87.4	3.4	9.2	
*P. citrinum*	KI	96.2	3.8	0.0	[10]
	KIO_3_	96.1	3.9	0.0	
*P. roquefortii*		88.0	1.3	0.46	[2]
*T. viride*	KI	83.6	3.0	13.5	This study
	KIO_3_	76.9	3.2	19.9`	

**Table 5:** Changes in extracellular organic acid production in response to iodine species.

Species	Treatment	Acetate	Citrate	Gluconate	Itaconic Acid	Lactate	Malate	Oxalate	Sorbate	Succinate	Tartrate
*A. fischeri*	KI	+1083.37 ± 23.41 ***	ND	ND	ND	+13.92 ± 1.43	ND	ND	−0.82 ± 0.60	ND	−1.21 ± 0.02
	KIO_3_	+26.26 ± 12.91	ND	ND	ND	+8.29 ± 0.45	ND	ND	+18.85 ± 2.59	ND	−1.63 ± 0.04
*A. terreus*	KI	−17.37 ± 15.28	ND	−18.26 ± 11.00	+26.21 ± 4.10	ND	+5.00 ± 0.18	+65.22 ± 40.94	−52.78 ± 20.29 *	+8.89 ± 0.12 **	+10.28 ± 0.46
	KIO_3_	+24.85 ± 9.52	ND	+4.39 ± 0.89	−6.82 ± 1.59	ND	+4.48 ± 0.52	+17.09 ± 2.90	+17.49 ± 4.17	+4.72 ± 0.66	+1.05 ± 0.20
*F. poae*	KI	+29.21 ± 4.40	ND	ND	ND	+7.62 ± 0.39	−61.09 ± 43.27	ND	+56.72 ± 6.59	ND	−6.19 ± 0.54
	KIO_3_	+12.82 ± 2.45	ND	ND	ND	+16.58 ± 2.32	+92.45 ± 64.54	ND	+75.66 ± 12.23	ND	+7.08 ± 1.55
*P. chrysogenum*	KI	+39.79 ± 2.24 **	−21.27 ± 4.57	+16.41 ± 1.40	ND	+10.44 ± 1.74	ND	ND	ND	ND	+4.35 ± 0.42
	KIO_3_	−31.23 ± 13.55	−11.12 ± 5.81	+9.32 ± 4.32	ND	−40.29 ± 17.32	ND	ND	ND	ND	−38.80 ± 14.10
*A. tenuissima*	KI	−5.65 ± 1.03	ND	+21.89 ± 1.79	ND	+7.00 ± 0.39	ND	ND	+0.12 ± 0.04	ND	+2.90 ± 0.23
	KIO_3_	−13.79 ± 3.62	ND	−16.38 ± 1.33	ND	+10.33 ± 0.96	ND	ND	−17.04 ± 11.32	ND	−44.58 ± 38.78
*T. viride*	KI	−100	+5.32 ± 3.40	ND	ND	−2.80 ± 1.32	ND	ND	−14.96 ± 7.81	ND	−20.32 ± 15.46
	KIO_3_	−100	−70.81 ± 57.81	ND	ND	−8.12 ± 1.53	ND	ND	−3.70 ± 0.38	ND	−28.38 ± 2.43

Percentage values (%) indicate relative increases or decreases in metabolite concentrations compared to the corresponding control samples cultivated without iodine addition. Statistical significances: *** *t* < 0.001; ** *t* < 0.01; * *t* < 0.05.

## Data Availability

All available data are presented in this article.
